# Personalized Medicine for Antibiotics: The Role of Nanobiosensors in Therapeutic Drug Monitoring

**DOI:** 10.3390/jpm10040147

**Published:** 2020-09-25

**Authors:** Vivian Garzón, Rosa-Helena Bustos, Daniel G. Pinacho

**Affiliations:** 1 PhD Biosciences Program, Universidad de La Sabana, Chía 140013, Colombia; viviangaru@unisabana.edu.co; 2 Therapeutical Evidence Group, Clinical Pharmacology, Universidad de La Sabana, Chía 140013, Colombia; rosa.bustos@unisabana.edu.co

**Keywords:** biosensors, therapeutic drug monitoring (TDM), antibiotic, personalized medicine

## Abstract

Due to the high bacterial resistance to antibiotics (AB), it has become necessary to adjust the dose aimed at personalized medicine by means of therapeutic drug monitoring (TDM). TDM is a fundamental tool for measuring the concentration of drugs that have a limited or highly toxic dose in different body fluids, such as blood, plasma, serum, and urine, among others. Using different techniques that allow for the pharmacokinetic (PK) and pharmacodynamic (PD) analysis of the drug, TDM can reduce the risks inherent in treatment. Among these techniques, nanotechnology focused on biosensors, which are relevant due to their versatility, sensitivity, specificity, and low cost. They provide results in real time, using an element for biological recognition coupled to a signal transducer. This review describes recent advances in the quantification of AB using biosensors with a focus on TDM as a fundamental aspect of personalized medicine.

## 1. Introduction

The discovery of antibiotics (AB) ushered in a new era of progress in controlling bacterial infections in human health, agriculture, and livestock [1] However, the use of AB has been challenged due to the appearance of multi-resistant bacteria (MDR), which have increased significantly in recent years due to AB mismanagement and have become a global public health problem [2]. More than 70% of bacteria are resistant to all or some of the known AB [3], creating the need for the development of new types of AB or the use of antimicrobial therapies with highly toxic “last-line” drugs to achieve effective treatment, mainly in critically ill patients [3]. According to the literature, it is estimated that, by 2050, antimicrobial drug resistant infections could kill 10 million people worldwide each year and cost around USD 100 trillion without the production of new molecules [3,4].

In order to stop this growing number of MDR infections, the World Health Organization (WHO) has established a series of measures among which are controls on the sale, administration, and dosage of AB [5,6]. Due to the fact that currently most doses are uniformly delivered to patients without taking into account the progress of the infection and the clinical picture, treatment failures are generated, which could lead to sub-therapeutic or toxic doses [2,7,8]. Among the solutions is the implementation of therapeutic drug monitoring (TDM), which can quantify drugs with a narrow therapeutic index (TI) that have high toxicity by tracking pharmacokinetic (PK) changes [6,9].

Monitoring techniques include single or mass-coupled chromatographic methods with a variety of detectors, including ultraviolet or fluorescent detectors (specified below) and immunoassays [10,11]. Many of these techniques have been approved by the Food and Drug Administration (FDA) of the United States [12]. However, they are expensive techniques that require specialized laboratories and trained personnel. An innovative solution to this problem is the use of nanobiotechnology, specifically biosensors that allow the measurement of drugs in body fluids (especially in blood, plasma, serum, and urine) [13]. These have become a new alternative as a specific, sensitive, and low-cost devices that can be miniaturized to be taken to the patient’s bedside and easily operated by doctors or health personnel [14,15].

The main advantages of biosensors are a low sample volume, minimally invasive methods for sample collection, reduced reagent consumption, short analysis time, multiple analyte detection, and portability [16,17]. These features make them a new alternative for individualized therapy in real time, allowing timely decision making [18]. Therefore, health and economic sectors are benefiting of the use of nanobiosensors, reducing the residence times of patients in hospitals, lowering the cost of treatments, and reducing MDR strain infections that generate high cost on health systems worldwide [17,19,20]. According to this, monitoring with biosensors is a tool with many advantages, and this kind of devices will become indispensable equipment in clinical use, contributing to the reduction of hospital costs of the health system [21].

Electrochemical and optical biosensors are the most used types of biosensors for the quantification of AB in different matrices. These have been used in the quantification of vancomycin [22], tobramycin, doxorubicin [23], and kanamycin [24], among others in different matrices with low detection limits in the microgram to nanogram range. This article reviews the use of biosensors for the quantification of antibiotic molecules in different matrices and their use in TDM as an alternative way to generate data when establishing a dose focused on a patient’s personalized medicine, minimizing the adverse effects inherent in the treatment.

## 2. Antibiotics

The revolution in the treatment of infectious disease started in 1929 with the discovery of penicillin, a molecule capable of inhibiting the growth of some pathogenic bacteria among which are *Staphylococcus*, *Streptococcus*, and *Pneumococcus* [25]. This fact was a turning point, since it helped to save incalculable human lives during World War II, being one of the biggest medical discoveries in the fight against infections worldwide [4,26]. The AB include a set of heterogeneous molecules with different PK and pharmacodynamic (PD) behaviors, which are characterized by having a specific action on cellular targets present in bacterial cells, inhibiting or stopping their growth [27].

Over time, the discovery of new antibiotic molecules from microorganisms (mainly actinomycetes and fungi) or synthetically obtained (sulfonamides and quinolones) has grown exponentially [28], and AB are widely used worldwide in clinical, veterinary, and agricultural fields [1]. However, despite the great advantages and development of AB over time, these molecules have been mishandled, putting at risk millions of lives [29]. This is attributed to the fact that bacteria have developed multiple resistance mechanisms against most AB, which prevents a favorable response to severe infectious conditions that are mainly caused by multi-resistant bacteria (MDR) [29,30]. At the same time, the misuse of antibiotics has generated economic issues both in the health system and in the pharmaceutical sector [31], mainly due to the high hospital costs of patients in intensive care units (ICU) and the low investment by some pharmaceutical companies in the development of new antibiotic molecules [32,33]. The few discoveries of new molecular entities with antimicrobial properties is mainly due to the low return on investment, the short time to the death of patents and, therefore, short treatment times, the increase in legal requirements, and the reserved use of new drugs for certain patients or special situations [31,34,35]. An example of this is the decreasing number of new molecules coming in to the market; since in 1998 there were 20 new antibiotic molecules, while despite the high degree of resistance in 2011, only four new antibiotics were reported [36]. In addition, a report from Medina in 2014 revealed that pharmaceutical companies, such as Abbott, Merck, and Roche had significantly reduced the investment for new antibiotics development. Astra-Zeneca, GlaxoSmithKline, Novartis, Sanofi-Aventis, and Schering-Plough are the only pharmaceutical companies that continue researching for new AB molecules [34,35].

This scenario is less and less encouraging, as pointed out by Nielsen and collaborators in 2019 reporting growing concern worldwide. Although the number of new antibiotics approved by the FDA has increased in the last six years [37], most of them do not meet the needs of patients and are redundant, since they share chemical structures and sites of action with former antibiotic molecules, which are already ineffective due to multiple resistance modalities developed by their target microbes [38,39]. For this reason, the WHO has been implementing sustained plans for development of new antibiotics, with the aim of increasing research on new molecules that can help mitigate resistance. However, this has not yielded the expected results so far [36].

It is estimated that at least 480,000 people will develop an MDR bacterial infection every year, if necessary measures are not taken to minimize resistance, which could lead to death due to the toxicity of last-line AB, the lack of new molecules, and poor accessibility to healthcare in developing countries [40]. Moreover, antimicrobial resistance (AR) compromises the effectiveness of treatments against diseases, such as HIV and malaria, among others [5]. This situation means that the number of AB used for MDR is increasingly reduced, and health personnel are forced to use some molecules that have high toxicity, such as colistin [41]. Research of new antibiotic alternatives is not an easy task due to the genotypic and phenotypic characteristics of the microorganisms to be inhibited, the PK and PD characteristics of patients, and the high costs of producing new molecules as result of the increase in regulatory requirements [42]. Thus, the development of novel AB has become a highly complex challenge [29].

As a consequence, one of the most effective solutions is the monitoring of some antibiotic molecules [43], with a view toward personalized medicine that reduces the risks inherent in AB treatment, such as toxicity, and allows the decrease in infections by resistant strains due to the individualization of the dose [9]. To achieve this, there are different techniques that detect or quantify AB in body fluids [44]. These results have generated a more practical and safer scenario for the treating doctor.

### 2.1. Classification of Antibiotics

There are a large number of reported antibiotic molecules with different characteristics that allow them to be classified into different groups. Many antibiotic molecules have been discovered with different targets in the bacterial cell, depending on the morphological characteristics of the microorganism [45]. To reach intracellular targets, molecules must cross the cell wall (depending on the nature of the antibiotic). For hydrophilic molecules less than 600 Da, this is achieved by proteins called porins, while in case of lipophilic AB, it is necessary to transport them with energy consumption or via diffusion through the lipid membrane [45]. Once inside the cell, AB must avoid inactivation and effectively recognize their pharmacological target. Gram-negative bacteria (GNB) are more resistant than Gram-positive bacteria (GPB) due to the presence of an external lipid membrane that surrounds the GNB wall, which prevents the passage of some molecules. However, there are some exceptions when the pharmacological target is the outer envelope [46].

**Figure 1:**
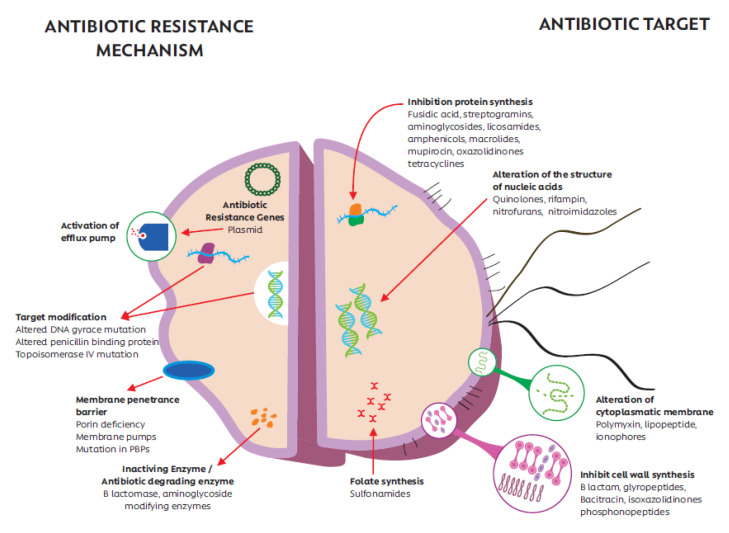
Main modes of action of antibiotics (AB) and antimicrobial resistance (AR) mechanisms of bacterial cells.

The most widely used classification of AB is based on molecular structure, mode of action, or spectrum [47]. AB generally show similar effectiveness and side effects when they have similar structural characteristics and mechanisms of action [48]. According to the mode of action, AB are classified mainly depending on (i) ability to inhibit bacterial wall synthesis, (ii) alteration the cytoplasmic membrane, (iii) inhibition protein synthesis, (iv) alteration the metabolism or the structure of the nucleic acids, (v) blocking the synthesis of metabolic factors, and (vi) inhibition β-lactamases. Figure 1 shows the main modes of action of AB on bacterial cells. [49]

#### 2.1.1. Inhibition of Bacterial Wall Synthesis

The cell wall is a membrane that covers the bacterial cell, characterized by supporting the internal osmotic pressure of the cell and allowing communication with the outside [49]. In order for the AB to act on the wall synthesis, the bacteria must be in the exponential phase and in an isotonic or hypotonic medium that favors cell lysis [50].

Three stages are involved in wall synthesis, which are targets for the different AB. *Cytoplasmic phase inhibitors*: In general, this type of AB inhibits or alters the precursors involved in wall synthesis [26]. Within these are two groups of AB, isoxazolidinones and phosphonopeptides [51]. *Inhibitors of the precursor transport phase through the cytoplasmic membrane*: Bacitracin is the most relevant group for this mechanism, which inhibits the incorporation of amino acids and nucleotides in the cell wall [52,53]. *Inhibitors of the structural organization of peptidoglycan*: At this stage, the peptidoglycan precursors are assembled with the help of penicillin-binding proteins (PBP), which have transpeptidase, transglucosylase, and carboxypeptidase activities, allowing them to interlock peptidoglycan components to form the bacterial wall. AB involved in this mechanism include beta-lactams and glycopeptides [54].

#### 2.1.2. Disruption of the Cytoplasmic Membrane

Polymyxins and lipopeptides belong to this group and are molecules capable of altering the structure of the bacterial membrane, changing its permeability and generating influx or efflux of ions that can lead to cell death [55]. The initial target of polymyxin is the lipopolysaccharide (LPS) component of the outer membrane (OM) in GNB, which is able to form aggregates and is responsible for the permeabilizing action of OM [56]. In the case of lipopeptides (daptomycin has been the most studied), the AB interacts with the lipid tails of the membrane and is partially inserted. In the presence of Ca^2+^, anionic daptomycin binds to the anionic groups of the lipids of the bacterial membrane, and is thus able to insert more deeply into the membrane and form aggregates, perforating the membrane, causing depolarization or some other event associated with the membrane [57]. These AB are characterized by being highly toxic, due to the similarity between prokaryotic and eukaryotic cytoplasmic membranes [50].

#### 2.1.3. Inhibition of Protein Synthesis

Protein synthesis is one of the most common target mechanisms of AB, because there are considerable differences between prokaryotic and eukaryotic systems. Among the main groups are mupirocin, a molecule capable of inhibiting the activation phase of proteins, preventing the incorporation of isoleucine into peptides [58,59]. Other families involved in the inhibition of protein synthesis are oxazolidinones, aminoglycosides, tetracyclines, amphenicols, lincosamides, macrolides, streptogramins, and fusidic acid; molecules capable of inhibiting the onset of protein synthesis and preventing the formation of complexes between translation elements (30S subunit, mRNA, initiation factors, and formylmethionyl-tRNA). The inactivation of the initiation complex or the inhibition of the elongation of protein lead to cell death [60,61,62,63,64,65,66,67].

#### 2.1.4. Antibiotics that Act on the Metabolism or Structure of Nucleic Acids

A series of intermediaries and enzymes are involved in the processes of replication, transcription, and translation of nucleic acids. Some AB groups have action on this intermediaries and enzymes, such as quinolones, rifampicin, nitroimidazoles, and nitrofurans [68,69].

Quinolones act on chromosomal DNA by binding to topoisomerases (specifically topoisomerase IV) and gyrase by inhibiting their action [70]. In GNB bacteria, quinolones mainly inhibit DNA gyrase, interacting with the amino acids of the α-helices close to the tyrosine present in the active center that are involved in the separation of the DNA chains [71]. In GPB, AB molecules act on topoisomerase IV by breaking DNA strands after each replication in addition to having a relaxing effect on the chain [72]. Rifamycins are characterized by inhibiting the synthesis of ribosomal and messenger RNA chains, because they bind to the β subunit of RNA polymerase, preventing the start of transcription [26,73]. The most commonly used antibiotic in the clinic is rifampicin [74]. Nitroimidazoles generate radicals that affect DNA chains; they are mainly used against anaerobic microorganisms, which has allowed them to be used both in the clinic and in veterinary medicine [75,76,77]. Nitrofurans, like the previous molecules, are compounds that are reduced in the bacterial cytoplasm, forming toxic compounds causing damage DNA structures. At the same time, they inhibit acetyl coenzyme A, interfering in the carbohydrate metabolism and inhibiting cell-wall synthesis [78]. However, its mechanisms of action have not been widely studied. An antibiotic with high clinical relevance is nitrofurantoin, which is used against both GPB and GNB bacteria [79].

#### 2.1.5. Blocking the Synthesis of Metabolic Factors

Bacteria require folic acid to carry out different metabolic processes essential for their survival, i.e., to obtain essential compounds, such as amino acids or puric bases. The AB of the sulfamide and diaminopyrimidine groups are capable of inhibiting folate synthesis pathways, leading to cell death [80].

The growing demand for AB has driven the search of strategies that allow the discovery of new molecules. One of them is the use of directed AB based on the search for biosynthetic genes (BGC) encoding secondary metabolites by the use of bioinformatic tools to determine the position of genes and the production of new molecules [81]. Investigations carried out by Juretic et al. in 2009 reported the synthesis of highly selective antimicrobial peptides through the use of computational tools. Starting from frog-derived peptides, a 23 residue glycine-rich peptide was synthesized, called adepatin 1, with bacterial activity against *Escherichia coli* [82]. Another strategy in the search of new AB consists in the use of enzyme assembly line engineering, although it has been a highly debated technique for years, due to its low efficacy. There are also new ways to create structures with improved properties based on existing ones. This has been shown to be effective against certain microorganisms [83]. Studies have also been conducted with antibacterial monoclonal antibodies directed towards exotoxins or endotoxins. These have been approved by the FDA for use in humans in the treatment of *Bacillus anthracis* (raxibacumab and obiltoxaximab) [84,85] and *Clostridium difficile* (bezlotoxumab) [86] in adult patients.

In recent years, a small number of new AB molecules have been discovered, which are at different stages of development. Antibiotics approved from 2015 to date in the United States and the European Union are ceftazidime/avibactam, ceftobiprol, ceftolozane/tazobactam, dalbavancin, oritavancin, solithromycin, and tedizolid. Drugs in phase 3 trials include carbavance, cadazolid, iclaprim, and finafloxacin, among others [87,88]. However, despite the PD of AB and their bactericidal or bacteriostatic action on bacteria, microorganisms have developed strategies that give them the ability to develop AR mechanisms, making their elimination increasingly difficult.

### 2.2. Issue of Bacterial Resistance to Antibiotics

Bacteria have developed different AR mechanisms in order to survive these agents and be able to colonize tissues [89]. AR is linked to natural phenomena including genetic recombination, genome dynamics and adaptation, leading to most of bacterial species being resistant to more than one AB molecule [42]. The main AR mechanisms involve the production of enzymes for chemical structure of AB, and among these, the presence of flow pumps, membrane alterations, modification of the target site, and horizontal transfer of resistance genes stand out (Figure 1) [90,91,92,93]. It should be noted that horizontal transfer is a selective advantage of prokaryotes, which has allowed the accelerated increase in MDR. This property allows the acquisition of different resistance mechanisms present in genes on plasmids that are transferred from bacteria to bacteria, making this characteristic one of the main mechanisms of AR to AB [94,95,96]. Table 1 shows the main mechanisms of AR and the genes involved in bacterial resistance.

As mentioned above, AR mainly arises due to poor management of AB and has become a public health problem that requires urgent action [42]. An example of this fact is the increasing number of acute respiratory infections cases, diarrheal diseases, and tuberculosis, among others. These pathologies cause more than 85% of deaths from infection in the world, due to the fact that most of them are caused by MDR bacteria [97]. These types of microorganisms spread easily due to the accelerated increase in globalization, leading to high rates of contagion worldwide [98]. This also has an impact on the healthcare cost throughout the world, as a result of the ineffectiveness of treatment, which in the worst-case scenario leads to death. Taking into account that health costs are not covered likewise for developed and underdeveloped countries, the latter do not have the same access to resources, and the use of new molecules is limited or absent [99].

The main causes of increased AR are general misinformation of patients, which has led to self-medication and/or mismanagement of the doses prescribed by doctors. This misuse of AB, without taking into account the PK and PD properties of the molecules [100,101], generate the appearance of new strains resistant to most of the known AB. Other factor that influences the acquisition of AR by bacteria is the constant contact of bacterial strains with traces of AB from the effluents of pharmaceutical companies and hospitals, where high concentrations have been reported in magnitudes of g/L [102]. Another source of AR is the presence of traces of AB reported in the agricultural and livestock sector, since AB are used as growth promoters in animals. This practice leads to variable concentrations of AB in meat, milk, and manure; the latter often used as organic fertilizer for crops [103,104,105]. Additionally, AB are administered to animals at high therapeutic doses to control infections, and on many occasions, they are applied in a generalized way without taking into account the specific strain of the microorganism or the physiological and general health condition of the animal. Considering the above and as a result of these activities, Ye et al. in 2007 reported the presence of traces of sulfonamides, macrolides, and quinolones in concentrations of ng/L in drinking water, groundwater, and surface water [106,107]. Therefore, the misuse of AB has been translated into an increase in MDR bacteria, making this issue one of the biggest public health concerns of the 21st century, according to the WHO [108].

According to the WHO, more than 50% of bacteria, such as *Escherichia coli, Klebsiella pneumoniae*, and *Staphylococcus aureus,* are reported to be resistant to AB often used around the world [40]. In 2008, a first list of six pathogens was created under the tittle “ESKAPE pathogens” that includes bacteria, such as *Enterococcus faecium, S. aureus, K. pneumoniae, Acinetobacter baumannii, Pseudomonas aeruginosa*, and *Enterobacter* with a high MDR [109]. In 2014, the United States FDA presented a final list of 21 target pathogens with a high unmet medical need [110]. It should be noted that this list shows that AR is not a problem restricted only to clinical care, but that community-acquired infections such as urinary tract infections, gonorrhea, and tuberculosis are mainly increased by MDR or extremely drug-resistant (XDR) bacteria [111].

Recent data show that 63% of *Acinetobacter* isolates, 13% of isolated *Pseudomonas*, and 11% of isolated *Klebsiella* causing health-associated infections in the United States are MDR [112]. According to the SENTRY (Antimicrobial Surveillance Program) study, Latin America shows higher levels of antimicrobial resistance than other evaluated regions, such as the United States of America and Europe. In South America, a high prevalence of extended-spectrum beta-lactamases (ESBL)-producing *Klebsiella pneumoniae* (between 45.4 and 51.9%) and *Escherichia coli* (between 8.5 and 18.1%) has been reported [113]. Mortality from methicillin-resistant *Staphylococcus aureus* (SAMR) is 2.5 times higher than from methicillin-sensitive *Staphylococcus aureus* (SAMS) [97]. For all these reasons, WHO has determined that AR would be a potential cause of death for 300 million people over the next 35 years and would have a high impact by decreasing gross domestic product by 2–3.5% compared to what it could be by 2050 [114]. With these predictions based on high uncertainty, economic and health sectors should be concerned with AR. Most regions of the world consider AR as a future threat to the growth and development of nations [5].

Latin America is one of the regions with most nosocomial outbreaks by AR, accounting for millions of lives in the last 60 years, making it one of the three most dangerous factors for public health in the 21st century [2]. Studies carried out by the Pan American Health Organization (PAHO) determined that, in Latin America and the Caribbean, AB are dispensed without medical prescription, according to a survey carried out in these countries, declining world health outlook, and favoring bacterial resistance [115].

This situation has led the WHO to conclude that the world is running out of AB. The multiple new AR mechanisms are spreading and are increasing the cost of healthcare, mainly due to the long stays at hospitals of patients suffering from MDR and XDR infection [116]. According to the WHO, bacteria resistant to more than two AB have been isolated from the same AB family, or from different families. These include carbapenem-resistant *Acinetobacter baumannii*, carbapenem-resistant *Pseudomonas aeruginosa*, vancomycin-resistant *Enterococcus faecium*, methicillin-resistant *Staphylococcus aureus* with intermediate sensitivity and resistance to vancomycin, campylochrine-resistant *Helicobacter pylori* spp. resistant to fluoroquinolones, *Salmonellae* resistant to fluoroquinolones, *Neisseria gonorrhoeae* resistant to cephalosporin and fluoroquinolones, *Streptococcus pneumoniae* without sensitivity to penicillin, *Haemophilus influenzae* resistant to ampicillin, and *Shigella* spp. resistant to fluoroquinolones [110,117].

To control the indiscriminate use of AB, strategies have been implemented to mitigate the impact of MDR on patients and economy, such as social campaigns by health professionals focused on the regulation and proper use of medications, which have been largely ineffective [115]. At the same time, PAHO has created an antibiotic surveillance network, studying the susceptibility of some bacteria to AB in order to take actions for continuous improvement and good management by clinical personnel [118]. Highly toxic or narrow therapeutic window antimicrobials, such as aminoglycosides, glycopeptides, and some β-lactams (e.g., carbapenems), are being used [119,120], characterized by high nephrotoxicity, neurotoxicity, or muscular toxicity. However, at present, medical personnel has been forced to treat some patients with these drugs as an effective way for growth inhibition the of pathogenic bacteria [121].

Therefore, in order to avoid adverse reactions from this type of drug, it is necessary to measure the levels of AB with narrow therapeutic window, leading to personalized medicine that could have a high impact in public health and global economy. Nowadays, therapeutic drug monitoring (TDM) has become a fundamental tool to control AB doses in critically ill patients, increasing the chances of overcoming MDR bacterial infections. In this sense, nanobiotechnology is an innovative solution for mitigating the antimicrobial resistance. In particular, the use of nanobiosensors for AB detection in plasma of patients allow the real time quantification on site, using a low sample size easily and quickly [14,15,22]. These point of care devices are a helpfully tools for clinicians to decision making [24], reducing the impact of infections by MDR strains and, therefore, contributing to control this worldwide public health issue [23,24].

## 3. Therapeutic Drug Monitoring (TDM)

**Figure 2:**
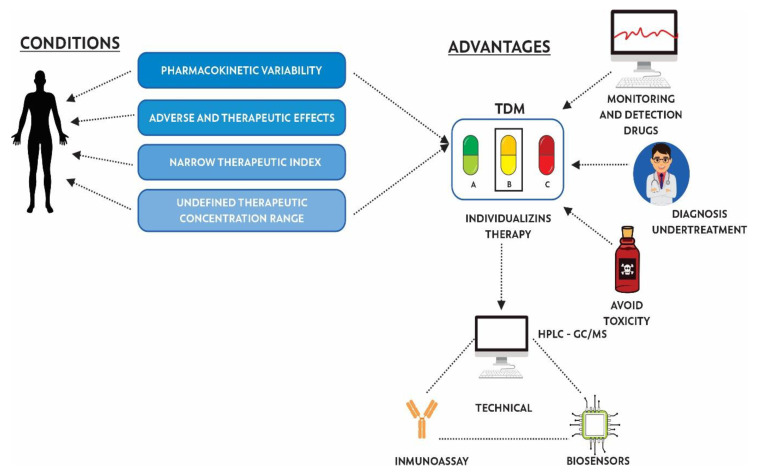
Conditions, advantages, and techniques used for the implementation of therapeutic drug monitoring (TDM).

TDM has been used since the early 1970s to personalize pharmacotherapy, with the aim of individualizing the dose of a drug, keeping drug concentrations in body fluids within a target range, minimizing adverse effects on the patient, and helping health personnel to determine the correct dose [160]. TDM helps to decrease PK variability (effects caused by the organism on the drug, in terms of absorption, bioavailability, distribution, metabolism, and excretion) and PD variability (effects of the drug on the organism, studying receptor binding and chemical interactions) [161]. According to WHO reports, certain criteria allow for determining whether a drug needs to be monitored, including: (i) pharmacokinetic variability, (ii) adverse and therapeutic effects related to concentration, (iii) narrow therapeutic index, (iv) undefined range of therapeutic concentration, and (v) difficult to control desired therapeutic effect (Figure 2) [6,10].

These criteria allow for understanding drug interactions in the body, so they are useful in cases where it is not clear whether the correct medication is being administered, if drug–drug interactions are occurring, or if poisoning is occurring [7]. According to the WHO, the drugs that require monitoring are AB (aminoglycosides and glycopeptides, β-lactams, fluoroquinolones, oxazolidinones, lipopeptides, and polymyxins), anticonvulsants (valproic acid, phenytoin, phenobarbital, and carbamazepine), cytotoxic drugs (methotrexate), antiarrhythmics (digoxin), and immunosuppressants (cyclosporine), because they are indispensable drugs for the treatment of a large number of diseases in today’s clinic [162].

At the same time, TDM has had a positive impact in economic terms, since it has allowed reduce the costs of hospitalization of patients through an effective dose of the correct medication, gradually eliminating the triggering of future related pathologies. It also allows clinicians to use drugs with a narrow therapeutic window [7].

### 3.1. Therapeutic Drug Monitoring of Antibiotics as Personalized Medicine

Most drug dosages were defined in healthy adults during the drug development phase [11] by assessing the condition of a selective group of patients. However, these characteristics are not representative of all the patients who use these drugs, due to PK variability [12]. The “one dose for all” strategy is a mistake; it is the cause of death of some patients in health centers [163]. For correct managing the adverse effects of antibiotics in patients, a strategy directed towards personalized medicine is being implemented, which allows for determining the dose of the drug depending on the genetic, physical, and clinical condition of the patient, by means of assessing the concentrations of the drug in different body fluids [164].

TDM in the management of AB is mainly used to find a personalized dose that allows successful antibiotic therapy, low probabilities of AR, and minimizes side effects in patients as much as possible [120]. The main groups of AB reported in the literature that require TDM are aminoglycosides, glycopeptides, β-lactams, fluoroquinolones, oxazolidinones, lipopeptides, and polymyxins. The PK and PD variability of each patient must be taken into account, and TDM could become necessary for some AB for which this is not routinely performed, since they can represent a risk to life, as observed in Table 2.

Regarding the groups reported in the literature, aminoglycosides induce nephrotoxicity, ototoxicity, and neuromuscular blockage due to the presence of a positive charge at physiological pH [120]. Nephrotoxicity is mainly due to the fact that aminoglycosides are excreted by glomerular filtration, unmodified, and toxicity is mainly due to the absorption of AB in the epithelial cells of the proximal renal tubules after filtration, causing accumulation of AB, generating morphological and functional problems [165]. The toxicity incidence data range between 5 to 25% of treated patients and is related to the duration of administration and the dose. On the other hand, ototoxicity is mainly manifested by cochlear or vestibular damage and in most cases is irreversible if it is not detected in time [120].

The AB classes (e.g., fluoroquinolones, aminoglycosides, lipopeptides) show different measures of exposures, such as the peak concentration, AUC, and AUC/minimum inhibitory concentration (MIC). These parameters are correlated with the PK/PD modelling to aid the dose selection and dose optimization of antimicrobial agents. In this way, TDM provides a higher possibility of clinical success [166]. The measurement in serum/plasma of AB is generally done by chromatography analysis (HPLC and UV), but other methods can be used, such as nanobiosensors and immunochromatography, which does not require specialized equipment or toxic solvents [167,168].

Thus, it is necessary to establish a dosage regimen in which AB concentrations are measured, especially in prolonged medication use [119], considering the minimum and maximum serum concentrations after the third dose. Thus, depending on the drug, average serum concentration ranges have been established in which the drug is toxic. For example, gentamicin is nephrotoxic at concentrations >0.5–2 mg/L [169], tobramycin at >1 mg/L [13] and amikacin at >5 mg/L [13]. Plasma levels higher than these, could affect the proper functioning of the body and even lead to death.

Another interesting case is the glycopeptides, including vancomycin and teicoplanin [120]. Vancomycin is generally administered when the bacteria causing the infection are resistant to other lower spectrum AB. Studies have shown that high plasma vancomycin concentrations increase the risk of ototoxicity and nephrotoxicity, so an average plasma concentration of 12 to 15 mg/L should be maintained [170,171,172]. In addition, recent research has shown that this drug increases the toxicity of other nephrotoxic AB, such as aminoglycosides, leading to the production of antibodies and causing thrombocytopenia and bleeding [173]. Regarding teicoplanin, the incidence of nephrotoxicity is lower than in patients treated with vancomycin, but it is recommended to perform TDM in order to achieve the effectiveness of the antibiotic with plasma concentrations of <10 mg/L [174,175].

In the case of β-lactams, these AB are still widely used in the clinical setting due to the wide therapeutic range and the fact that they are rarely toxic [176]. However, in recent decades, due to changes in the minimum inhibitory concentration (MIC) of microorganisms and clinical alterations in critically ill patients, the therapeutic window has been reduced, mainly in the intensive care unit ICU [177]. The main monitored β-lactams are carbapenems, such as meropenem. According to studies reported in the literature, toxicity has been reported in critically ill patients with special conditions, such as dialysis treatment [178,179,180]. Fluroquinolones are generally safe AB, but adverse effects are inherent in patients with previous pathologies related to the gastrointestinal tract, central nervous system, kidneys, and tendons [181,182,183]. Furthermore, its bactericidal activity increases as the concentration of the drug increases in serum, becoming 10 times the MIC [184]. This makes it possible to generate a risk of overdose or a subtherapeutic action of the drug [13]. According to this, it has become necessary to monitor this type of drugs depending on the patient’s condition.

Linezolid is an oxazolidinone with a recommended daily dose of 600 mg twice daily and does not require TDM under current guidelines [185]. In contrast, recent studies have shown that this dose does not reach the therapeutic range in a considerable number of critically ill patients or those with kidney failure [186,187]. This is mainly due to changes in protein binding or drug metabolism, leading to high variability in plasma concentrations [188]. Garrabau et al. determined that, according to the genetic status of the patient, linezolid can produce mitochondrial toxicity in blood cells and nerve fibers of the skin [189]. Thus, it is necessary to monitor this type of molecule in patients who are in the ICU [13].

On the other hand, daptomycin requires TDM depending on the patient’s condition, mainly in septic and critical states [190]. Recent studies report musculoskeletal toxicity in patients receiving the antibiotic along with statins [191,192], with the presence of rhabdomyolysis after the administration of this drug regardless of dose [193]. In addition, liver damage can occur due to elevations in serum creatinine phosphokinase [194]. In 2019, Raza and coworkers found what they called a “rarity among rarities”, but which is currently affecting many patients: daptomycin is capable of inducing eosinophilic pneumonia, which is caused by the detection of an antigen by alveolar macrophages [195].

Despite advances and the effort in the search for new molecules, research has focused on determining the PK parameters of some AB that had been discontinued due to adverse effects, such as colistimethate sodium (CMS), the prodrug of colistin, in order to find the optimal dose to maintain an adequate benefit-risk balance [196]. Currently, there is controversy regarding the dose of CMS. The FDA, for example, proposes a recommended dose for colistin in the range of 2.5 to 5 mg/kg, while researchers in Europe recommend doses between 50,000 to 80,000 IU/kg. The simple fact of having different units has caused confusion amongst clinicians and can lead to overdose, increasing the side effects, or underdosing with the development of greater resistance and mortality (Table 2) [197].

Despite the fact that TDM is used more frequently for AB with a narrow therapeutic window, the interest in using TDM is increasing due to the raising number of patients for whom the PK of the antibiotic is not defined, causing ups and downs when controlling the infection (e.g., critical illness, significant comorbidities, the elderly, and extremes of body size) [8,198,199]. This will allow us to take actions to reduce the inappropriate use of AB, in order to control the growing public health problems that MDR bacteria are generating. To find a solution to this problem, it is necessary to generate strategies that allow the proper use and administration of these drugs. One of these strategies is the correct dosage of the AB, considering that most doses are currently delivered uniformly to patients, without taking into account the progress of the infection and the clinical picture [14,200]. In recent years, laboratory techniques have been used for measurement and monitoring of AB molecules.

### 3.2. Antibiotic Quantification Methods

The quantification and monitoring of AB is based on the development of laboratory techniques, including high performance liquid chromatography (HPLC), gas chromatography mass spectrometry (GC-MS), immunoassays, bacterial-growth-inhibition-based assays, and biosensors. Chromatographic techniques, including HPLC, are the reference techniques, robust, and with a high specificity. However, they require trained personnel, specialized laboratories with necessary equipment, and reagents, as well as extensive and time consuming sample processing procedures. Therefore, it is not possible get results in real time and also, due to the high cost, most hospitals in underdeveloped countries do not have the resources to implementation. However, they have been widely used for the quantification of AB, including β-lactams, macrolides, glycopeptides, daptomycin, and meropenem, due to their robustness and ability to quantify molecules in different matrices [228,229,230,231,232,233].

Herregodts and collaborators in 2019 reported a novel technique consisting on a device able to determine piperacillin/tazobactam or meropenem concentrations in exhaled air in critically non-ventilated patients. This device, called ExaBreath^®^, retains the antibiotic molecules that are analyzed by mass spectrometry after a purification process [234].

On the other hand, immunoassays are based on the selectivity and affinity of an antibody for the antigen. They are cheaper techniques compared to chromatography but also require a laboratory, reagents, and trained laboratory personnel [235]. These techniques include enzyme-linked immunosorbent assays (ELISA), which are able to detect gentamicin and vancomycin in samples containing proteins with ranges of 2–500 ng/mL for gentamicin and 20–5000 ng/mL for vancomycin [236]. Fluoroquinolones, such as levofloxacin, have also been detected in urine using a fluorescence polarization immunoassay assay ranging from 2.5 to 50 ng/mL based on garenoxacin labeled with 4-aminomethylfluorescein and polyclonal antibodies (pAb) against levofloxacin [237] (Table 3). For an AB like colistin, pAb have been used for quantification, mainly in food [238,239]. In addition, tobramycin and kanamycin have been determined with a lower limit of quantification of 0.30 mg/L, verified in human plasma [240]. Due to the precision, selectivity, reliability, and low cost based on this technique there are several commercial kits and reagents on the market that allow the determination of some AB requiring TDM. Some examples of commercial kits include QMS^®^ Tobramycin (TOBRA), QMS^®^ Gentamicin (GENT), QMS^®^ Vancomycin, Monoclonal Antibody Penicillin (mAb) (P2B9), and ARK™ Linezolid assays.

In the case of bacterial-growth-inhibition-based assays, they can determinate the presence or absence of AB in patient or food samples. These tests are performed by inoculating dilutions of the body fluid or food samples on a bacterial culture sensitive to the administered drug [262]. This technique is inexpensive, and the presence or absence of AB in a sample is determined easily. However, it has low sensitivity and robustness, since it is limited by time and conditions of the culture medium [263,264,265].

Finally, an innovative solution to this problem is the use of nanobiotechnology, specifically biosensors, for the quantification of AB in samples of body fluids [15,266,267].

## 4. Nanobiosensors as Bioanalytic Applications in the Quantification of Antibiotics

In recent years, biosensors have become an interdisciplinary tool of great help in clinical diagnostic processes and in different industries, such as food and agriculture [268]. They are characterized by high sensitivity, selectivity, and reliability that ensure that the biosensor interacts exclusively with the compound of interest, minimizing background noise. In addition, these devices have a long lifetime and are simple to handle, portable, and can be automated and miniaturized. Regarding the sample, this method has a low cost of analysis, no complicated pre-treatment is required, and the analysis time is short [269,270,271,272,273]. These features make biosensors an attractive alternative for compound quantification.

According to International Union of Pure and Applied Chemistry (IUPAC), a biosensor is defined as “a device that uses specific biochemical reactions mediated by isolated enzymes, immunosystems, tissues, organelles or whole cells to detect chemical compounds usually by electrical, thermal or optical signals” [274]. In other words, biosensor means a compact analytical device that incorporates a biological recognition element closely associated with or integrated into a transducer that allows the processing of the signal generated by the interaction between the recognition element and the ligand [275]. According to that, biosensors are classified in terms of the nature of the biological component and the transduction system used [276].

The biological components are classified as biocatalytic or affinity. Biocatalytic components use biocatalysts in isolated enzymes or multi-enzyme systems, cell organelles, whole cells, or animal/plant tissues. The signal is based on the measurement of the products generated by the catalyzed chemical reaction between the enzyme and the substrate [277]. Affinity bioreceptors are based on the interaction between the analyte and the recognition element, generating an analyte-receptor complex, which is detected by labeling (enzymatic or fluorescent) or by monitoring the change of a physical–chemical property of the transducer. The most commonly used biological components are antibodies, nucleic acids, microorganisms, aptamers, and receptor proteins [278].

Regarding transduction system, it is the biosensor element that turns variations in physical or chemical properties produced by the interaction between the analyte and the ligand into a signal that can be amplified, stored, and recorded [275]. There are different types of transducers, including electrochemical (amperometric, potentiometric and impedimetric), optical (fiber optic, surface plasmon resonance (SPR), surface-enhanced Raman scattering and biosensors of total internal reflection fluorescence (SERS), piezoelectric (microbalances of quartz crystals), and nanomechanical (nanolevers) [279]. Depending the nature of the sample and the analyte-ligand interaction, it is possible to choose the appropriate device. Table 3 shows the biosensors types used in AB quantification.

### 4.1. Electrochemical Biosensors

Electrochemical biosensors measure the electrochemical change produced by the analyte–ligand interaction [280]. Depending the type of signal obtained, they are classified as potentiometric (electrical potential difference) [281], amperometric (current generated by reduction and oxidation of electroactive substances) [282], or impedimetric (changes in conductance) [283]. Electrochemical biosensors have been used for the quantification of aminoglycosides in blood serum with ribonucleic acid (RNA) aptamers with a detection range of 2–6 µM [241].

Amperometric biosensors have been used for the quantification of some AB molecules, such as penicillin G, by using gold nanoparticles (NP), with catalytic hydrolysis of AB with a low limit of detection (LOD) of 4.5 nM [242]. In addition, detection of chloramphenicol and kanamycin have been carried out with the use of antibodies as bioreceptors, with LODs of 45 µg/L and 6.31 μg/L, respectively [24]. This result is promising in contrast to LODs obtained by HPLC, where values of 38 mg/L have been reported [284]. The presence of amikacin in buffer solutions has also been determined using a carbon paste electrode modified with nano-sized copper oxide, obtaining an LOD of 0.58 µg/mL [243], compared to the LOD obtained by HPLC of 2.34 µg/mL [285]. Moreover, amperometric biosensors have been used in the food industry to quantify fluoroquinolones in milk, by combining modified magnetic beads with broad recognition profile antibodies for fluoroquinolones, with a haptenized enzyme and an electrode composed of magnetic graphite-epoxy (m-GEC). This was a highly reliable, fast, simple, and cost-effective device, with an LOD of 0.009 µg/L [244].

Regarding impedimetric biosensors, tobramycin, an aminoglycoside with a narrow therapeutic range, and several side effects, has been quantified in human serum using an RNA aptamer as a recognition element. The LOD obtained was 0.7 µM, concluding that detection of the antibiotic is limited by the dilution of the sample [245], in contrast with that reported by Shou et al., where the LOD for tobramycin in tissue fluid using the HPLC-MS/MS was 0.75 mg/L [286].

Impedimetric devices have been reported to detect AB in buffer, as is the case of label-free detection of ciprofloxacin based on the immobilization of anti-ciprofloxacin antibodies by chemical binding on a film of poly(pyrrole-NHS) electrogenerated on a solid gold substrate, showing an LOD of 10 pg/mL [246], lower than the obtained by HPLC in body fluids (0.05 μg/mL) [287]. Impedimetric biosensors have been used to quantify AB in different matrices, such as milk. An example of this is the detection of penicillin using a technology called Capture-SELEX (Systematic Evolution of Ligands by Exponential Enrichment). This technique is based on selection of deoxyribonucleic acid (DNA) aptamers using penicillin in solution, while a single stranded DNA (ssDNA) library is fixed on a support, providing an LOD of 0.17 µg/L [247]. Penicillin has also been quantified with a magnetic graphene nanoparticle (NP) nanocomposite (GR-Fe_3_O_4_NP) and a gold (PEDOT-AuNP) poly(3,4-ethylenedioxythiophene) NP compound, with an LOD of 0.057 ng/mL [248]. In addition, tetracyclines in milk have been quantified through the use of antibodies by testing an electrode based on oleic acid and modified carbon paste, which allowed a minimum LOD of 3.8 fM, showing high selectivity between different types of tetracycline [249].

Potentiometric biosensors have been used for the quantification of ofloxacin in urine and serum with an LOD of 10 μM. Further research determined that there is no significant interference from the excipients found in commercial formulations, nor in the different ions present in body fluids, allowing quantification without markers or interfering potentials [250]. As the above devices, potentiometric biosensors have been used in the detection of penicillin in foods, such as milk, with an LOD of 3 mM [288] or in the detection of sulfamethoxazole for environmental control [289].

### 4.2. Optical Biosensors

Optical biosensors are devices that detect changes in the properties of light, such as refractive index, absorption, fluorescence, or light scattering, as a result of the interaction between the analyte and the receptor [290]. These devices are grouped into two categories: bio-optrods and evanescent field-based sensors. The former are based on the interaction between the analyte and a reagent immobilized at the end of a fiber, producing a quantifiable change in the optical properties of the transducer, optically monitored by dyes, fluorescent, and biochemiluminescent molecules. Within these biosensors are fiber optic devices [291]. The evanescent field biosensors are based on the guidance of electromagnetic waves, transmitting light through internal reflections under conditions of total reflection, creating an evanescent field capable of penetrating certain distance from the surface of the waveguide modified with the receiver [292]. Within this type are included SPR devices, SERS devices, total internal reflection fluorescence (TIRF) biosensors, optical waveguide interferometric biosensors, ellipsometric biosensors, and spectroscopy biosensors employing reflectometric interference.

Fiber optic biosensors consist in a fiber where the recognition element is immobilized at the end. As a consequence of the interaction between the analyte and the recognition element, a change is generated in the marker, which spreads through the fiber to the detector [275]. This type of biosensor has been used in the quantification of tetracycline, oxytetracycline, and doxycycline with an anthracene-containing copolymer prepared from 9-anthrylmethyl methacrylate, methyl methacrylate, and n-butyl acrylate (PAMB). Detection is based on quenching of the fluorescence of the fiber due to the presence of the antibiotic in commercial and urine samples. The LODs are 0.1 μM for tetracycline and 2 μM for oxytetracycline and doxycycline [251].

This type of biosensor has also been reported for the quantification of antibiotic molecules in different matrices, such as foods or buffers, as in the case of doxorubicin and daunorubicin buffer solutions using LED fiber optics induced fluorescence, with LODs of 18 ng/mL and 13 ng/mL, respectively [23]. This method has also been used for the detection of moxifloxacin by hollow core photonic crystal fibers (HCPCF), with an LOD of 682.43 ng/mL in aqueous solution [252]. Korposh et al. have quantified vancomycin in porcine blood plasma enriched by means of long-term fiber optic grids functionalized with molecularly imprinted polymer NP (LPFG-MIP NPs), obtaining a very low LOD of 0.0032 ng/mL. This fiber optic sensor is capable of detecting target AB at low concentrations (~700 μM), even with traces of amoxicillin, bleomycin, and gentamicin in the medium [22]. In food sector, they have been very useful in detecting AB, such as sulfadimidine in dairy products, with an LOD of 0.05 ng/mL, using a portable and reusable optofluidic-based biosensing platform. This method has high sensitivity, portability, and acceptable reproducibility for the detection of sulfadimidine in real time in milk and other dairy products [253].

Surface resonance plasmon optical biosensors (SPRs) are based on an optical phenomenon generated when a polarized light beam is directed to a lower refractive index layer (metallic layer of gold or silver) between the prism and the sample. This light generates the excitation of a surface plasmon for a certain angle of incidence of said light, known as the resonance angle. The binding of the analytes with their recognition element induces a change in the refractive index and as a consequence the displacement of the resonance angle [275,293]. This type of biosensor has been used in different applications, such as in the clinic where tobramycin has been detected in patient serum based on a portable, palm-sized localized surface transmission plasmon resonance (T-LSPR) configuration. This device consist in standard components coupled with tobramycin-specific DNA aptamers, reaching a theoretical LOD of 3.4 μM, making it a portable, sensitive, and economical real-time measurement device [254]. Neomycin has been quantified by SPR using modified aptamers of ribonucleic acid (RNA), demonstrating that these aptamers can be modified to be resistant to enzymes, such as endonucleases, without variations in their analytical characteristics [294].

In addition to determination of AB in plasma samples, a large number of applications of SPR in the quantification of AB in food can be found in the literature. Faalnouri et al. have quantified amoxicillin in milk samples comparing a polymeric film (methacrylate hydroxyethyl-N-methacryloyl-(L)-glutamic acid) with similar polymer enclosing NP, finding LODs of 0.0012 ng/mL and 0.0009 ng/mL, respectively, according to the film, being a very sensitive technique for the detection of AB in milk [255]. Likewise, thiamphenicol, florfenicol, florfenicol amine, and chloramphenicol residues have been determined simultaneously in shrimp, using SPR with LODs of 0.1, 0.2, 250, and 0.5 ppb, respectively. These results show greater sensitivity with those obtained by an ELISA immunoassay [295]. The quantification of tetracycline hydrochloride and oxytetracycline hydrochloride in buffer solutions using a molecular printed polymer (MIP) has been reported based on various binding sites/nanocavities having the complementary form of the functional groups of the target molecules on its surface. This method raised LODs of 4.23 ng/mL for tetracycline and 4.05 ng/mL for oxytetracycline [296]. Likewise, traces of erythromycin in milk and honey have been quantified with LODs of 47.41 ng/mL and 28.48 ng/mL, respectively [256].

Sulfamethoxazole, an AB widely used for the treatment of infections and diseases in animals, such as chickens and cattle, has been quantified in the veterinary field. Detection was based on the SPR technique and functionalized carbon nanotubes (CNT), with an LOD of 225.98 ng/mL, lower than the corresponding to other techniques, such as Enzyme-linked Immunosorbent Assay ELISA [297]. In addition, neomycin has been quantified by molecular coupling with bovine serum albumin (BSA), demonstrating a strong interaction between the AB and protein at different concentrations (1–128 µM) using low density carboxymethyl dextran (CMD) modified gold surface chips [298]. Using the same method, other types of antibiotic molecules have been quantified, such as rifampicin, by immobilizing BSA on a carboxymethyl dextran hydrogel chip [298]. This is an alternative to the quantification of AB using SPR optical biosensors.

### 4.3. Surface-Enhanced Raman Scattering (SERS)

This type of biosensor is based on the intensity amplification of the Raman phenomenon by using metallic NP or metallic structures. When two particles get in contact and one of them has a rougher material on the surface, the electromagnetic field is dramatically amplified, resulting in a large amplification in Raman scattering [168]. There are few papers in the literature on the use of these devices in the clinic. One of them was carried out by Markina et al., where SERS was combined with liquid-liquid extraction in sulfamethoxazole-enriched urine, using silver NP stabilized with hydroxylamine as the SERS substrate, providing an LOD of 1.7 µg/mL. This would be quite useful to clinical staff, because this type of antibiotic is highly toxic when mixed with trimethoprim [257]. Furthermore, urine ceftriaxone has been quantified with the use of gold NP, with an LOD of 0.7 µM in a sample volume of 1 mL [258]. Moreover, Liu and collaborators quantified levofloxacin in mouse blood using a gold nanoparticle-coated fiber optic nanoprobe and SERS to measure levofloxacin lactate, representing a great opportunity to measure this type of AB in blood [299].

However, these types of devices have been used mostly in the food and environmental fields. For example, chloramphenicol has been detected in dairy products and honey by means of a polymer surface with a molecular impression, giving results in 15 min [300]. In addition, this technique has been used for the detection of ampicillin, penicillin G, carbenicillin, and penicilloic acid in deionized water by the use of hydroxylamine silver nanoparticles, with detection limits of 27, 29, 30, and 28 ng/mL, respectively. This is a promising methodology for the detection of different antibiotic molecules, including the penicillins, opening a new window to AB quantification [259]. Another application is the quantification of tetracycline in water samples using a Raman fingerprint strip sensor, coated with an anti-tetracycline mAb, obtaining an LOD of 0.04 ng/mL [260]. The use of gold nanoparticles and macroporous silicon provided high performance regarding the detection of the antibiotic with an LOD of 1 nM, compared to traditional techniques at pH between 5 and 6 [261].

This type of biosensor has also allowed the detection of quinolone residues in drinking water using silver NP and titanium dioxide (Ag-TiO2), with high detectability of difloxacin hydrochloride, ciprofloxacin, enrofloxacin, danofloxacin, and enoxacin, with LODs of 4.36, 70.8, 39.4, 31.6, and 315 pM, respectively. These concentrations are below the European Union (EU) maximum residue limit (3.01 × 10^−7^ mol/L) [301].

### 4.4. Piezoelectric (Quartz Crystal Microbalance)

Piezoelectric systems measure direct mass changes induced by the formation of the antigen–antibody complex (Ag-Ac). These devices consist on an oscillating crystal that resonates at a certain frequency when the interaction between the recognition element and the analyte takes place [275].

Most of these types of biosensors have been applied for the detection of AB in other fields different to clinic, such as food. Penicillin G and ampicillin have been detected in buffer using molecular printed nanoparticulate polymers (NMIP) as recognition element. LOD values of 0.04 µg/mL were found for penicillin G and 0.09 μg/mL for ampicillin [302]. Piezoelectric sensors have also been developed for the detection of β-lactams (penicillin G and cefotaxime) with LODs of 3.0 and 7.6 ng/mL, respectively, in meat and milk [302,303]. Furthermore, tetracycline molecules have been quantified by a molecularly imprinted polymer (MIP) with an LOD of 3 × 10^−7^ µg/mL [304].

### 4.5. Nanomechanical Biosensors

In this type of biosensor the biological recognition element is immobilized on the surface of a microlever, generally made of silicon, which is immersed in a liquid sample. The interaction between the analyte and the ligand induces a differential change in the surface tension of the liquid, which generates a change in deflection and/or in the resonance frequency [275]. Nanomechanical biosensors have been used in different areas, mainly in the identification of pathogens in human samples [305] and for the identification of proteins, such as topoisomerases or cancer marker proteins [306,307,308,309,310]. However, there is no evidence that this type of device was applied to the detection or quantification of antibiotics in samples of body fluids.

## 5. Conclusions

The increasing number of MDR infections constitutes a public health problem that is affecting the healthcare systems and the economy worldwide. This has occurred as a result of AB mismanagement, not only in the health sector, but also in agriculture, livestock, and the pharmaceutical industry and has led to the use of highly toxic molecules with narrow therapeutic indices. For this reason, strategies have been implemented, such as determining the correct dosage in patients by TDM, the detection of AB in foods (mainly in chicken, meat, milk, and honey) and quantification in effluents from pharmaceutical companies.

Biosensors are among the techniques used to quantify AB in different matrices in real time, at low cost and with highly reliable results. Since they are versatile, sensitive, and specific devices, they can be used on site and can be portable, providing advantages over conventional chromatography and immunoassay techniques, which are expensive techniques that require a specialized laboratory. The literature mainly reports on the use of biosensors in the quantification of AB in food for human consumption, as food is one of the main sources of traces of AB that lead to the generation of MDR. Biosensors represent a significant step toward the detection of AB in patient samples, in order to determine AB concentrations in patients in real time and, thus, provide personalized medicine by the means of TDM.

## Figures and Tables

**Table 1:** Involved genes in bacterial resistance.

Antibiotic	Resistance Genes	Mechanism of Resistance	Bacteria	Ref
***Aminoglycosides***				
**Gentamicin, neomycin, kanamycin, tobramycin, amikacin**	*aac (6′) - Ie + aph (2″)-Ia*	Encodes a bifunctional enzyme, AAC(6′)-APH(2”), that confers resistance to a broad spectrum of aminoglycosides, able to acetylate and phosphorylate antibiotics	*Staphylococcus aureus* *Enterococcus* spp., *Staphylococcus aureus, Streptococcus agalactiae* (group B), *Streptococcus mitis* and group G *Streptococcus*	[122,123]
**Neomycin, kanamycin, tobramycin, amikacin**	*ant(4′)-Ia*	Plasmid-encoded aminoglycoside nucleotidyltransferase	*S. aureus, S. epidermidis, S. aureus, Enterococcus* spp. and *Bacillus* spp.	[124]
**Neomycin, kanamycin**	*aph(3′)-IIIa*	Aminoglycoside 3′-phosphotransferase	*S. aureus, Enterococcus faecalis*	[125]
**Amikacin**	*aac (6′) - Ie + aph (3″)*	Modification of the amikacin molecule by acyltransferases	*Acinetobacter baumannii*	[126]
***Glucopeptides***				
**Vancomycin, teicoplanin**	*vanA*	Encodes a D-alanine-D-alanine ligase of modified specificity that synthesizes peptidoglycan precursors with reduced affinity for glycopeptide antibiotics	*Enterococcus faecium, Enterococcus faecalis, S. aureus*	[127]
**Vancomycin**	*vanB*	Synthesis of modified peptidoglycan precursors terminating in D-lactate.	*Enterococcus faecalis* V583	[128]
	*vanC*	Synthesize a dipeptide which is incorporated into peptidoglycan precursors	*Enterococcus gallinarum*	[129]
	*vanE*	Synthesis of modified peptidoglycan precursors	*Enterococcus faecalis*	[130]
	*vanD*	Synthesized peptidoglycan precursors terminating in D-lactate.	*Enterococcus faecium* BM4339	[131]
	*vanG*	The inducible synthesis of peptidoglycan precursors ending in D-alanine-D-serine	*Enterococcus faecalis* BM4518 *and* WCH9	[132]
	*vanM*	Encodes a D-alanine-D-alanine ligase of modified specificity that synthesizes peptidoglycan precursors with reduced affinity for glycopeptide antibiotics	*Enterococcus faecium*	[133]
	*vanL*	D-Ala-D-Ser, which is incorporated into peptidoglycan precursors, which subsequently have a low binding affinity for vancomycin	*Enterococcus faecalis* N06-0364	[134]
***Βeta-lactams***				
**Carbapenem**	*bla OXA-23*	Production of carbapenemases	*Acinetobacter baumannii*	[135]
	*blaOXA-24*			[136]
	*blaOXA-51*			[137]
	*blaOXA-58*	Carbapenem-hydrolyzing oxacillinase		[138]
	*bla KPC*	Production of carbapenemases	*Klebsiella pneumoniae, Enterobacteriaceae*	[139,140]
	*mexR*	Multidrug resistance operon repressor MexR	*Pseudomonas aeruginosa*	[141,142]
***Quinolones***				
**Fluoroquinolones**	*gyrA, gyrB, parC*, and *parE*	Alteration in enzymes, alterations in efflux pump activity	*Salmonella, K. Pneumoniae, A. Baumannii, P. Aeruginosa, Neisseria gonorrhoeae, Escherichia coli*	[143,144,145]
	*qnr*	Encodes a pentapeptide repeat protein that protects DNA gyrase from inhibition by fluoroquinolones	*K. pneumoniae, Enterococcus faecalis*	[146,147]
***Oxazolidinone***				
**Linezolid**	*cfr*	Methyltransferase activity	*Staphylococcus aureus, Enterococcus* spp.	[148,149]
***Lipopeptide***				
**Daptomycin**	*mprF*	Increase in the lysyl-phosphatidyl glycerol production	*Staphylococcus aureus*	[150]
	*yyG (walk)*	Synthesis of a histidine kinase sensor		
	*rpoB and rpoC*	Shown to cause cell-wall thickening and reduction in the negative charge of the outer layer		
	*cls2*	Encodes for a cardiolipin synthase		
	*agrA*	Encodes a quorum sensing system		
	*pgsA*	Synthesis of phosphatidyl glycerol		
	*pnpA*	Encodes for a polynucleotide phosphorylase		
	*dltABCD*	Involved in cell-wall teichoic acid D-alanination		
	*cls, gdpD*	Encoding enzymes of phospholipid metabolism	*Enterococcus*	[151]
***Polymyxin***				
**Colistin**	*arnBCADTEF* operon and *pmrE*	Modification of the lipid A with aminoarabinose	*Salmonella enterica, Klebsiella pneumoniae, Escherichia coli, Proteus mirabilis, Proteeae bacteria, Serratia marcescens and P. aeruginosa*	[41,152,153,154,155]
	*pmrAB, pmrD, phoPQ, parRS, mcr*	L-Ara4N and PEtn modification of lipid A	*E. coli, Salmonella enterica, P. aeruginosa*	[41,156]
	*Dlt-ABCD, graXSR, dra/dlt, liaSR*, and *CiaR* operons	Adding D-alanine (D-Ala) to teichoic acids, thereby increasing net positive charge	*Staphylococcus aureus, Bordetella pertussis, Streptococcus gordonii, Listeria monocytogenes* and Group B *Streptococcus*	[41,157]
	*siaD, cps operon, ompA, kpnEF, phoPQ*, and *rcs*	Loss of polymyxin target and capsule polysaccharide (CPS) overproduction	*Neisseria meningitidis, K. pneumoniae and S. enterica*	[41,158]
	*spgM, pgm, hldA, hldD, oprH, cj1136, waaF, lgtF, galT, cstII, galU*	Lipooligosaccharide (LOS) and LPS modification	*Salmonella typhimurium, Campylobacter jejuni* and *Haemophilus influenzae*	[41,158,159]

**Table 2:** Dose and maximum concentration (Cmax) of antibiotics (AB) that according to the pharmacokinetic (PK) characteristics require therapeutic drug monitoring (TDM).

Antibiotic	Adverse Effects	Dose	Cmax	Ref
**Aminoglycosides**				
**Gentamicin**	Nephrotoxicity, Neurotoxicity, Ototoxicity	5–7 mg/kg/day	5–10 mg/L	[201,202]
**Amikacin**		15–20 mg/kg/day	20–35 mg/L	[201,203]
**Tobramycin**		5–7 mg/kg/day	5–10 mg/L	[201,204]
**Glycopeptides**				
**Vancomycin**	Nephrotoxicity, Ototoxicity, Severe vesicular reactions, Hemorrhagic occlusive retinal vasculitis	15–20 mg/kg/12 h	20–50 mg/L	[170,171,205,206]
**Teicoplanin**	Nephrotoxicity, Ototoxicity, Thrombocytopenia		43 mg/L	[205,207,208]
**Polymyxins**				
**Colistin**	Nephrotoxicity, Neurotoxicity	150mg (single dose)	18 µg/mL	[209]
**β-Lactamics**				
**Penicillins**				
**Ampicillin-sulbactam**	Thrombocytopenia, eosinophilia, leukopenia, and transient elevation of transaminases	1000:500 mg	8–37 µg/mL	[210]
**Cephalosporins**				
**Cephalexin**	At high doses, coagulation disorders, platelet function disorders, leukopenias, thrombocytopenias, neutropenias, decreased hemoglobin and hematocrit, hemolytic anemias. Nephrotoxicity	0.25 g/6 h	14 µg/mL	[211,212,213,214]
**Cephradine**		0.5–2g/6 h	12 µg/mL	
**Cefoxitin**		1–2 g/6–8 h	20 µg/mL	
**Cefuroxime**		0.5–1g/6–8 h	40 µg/mL	
**Ceftazidime**		1–2 g/8–12 h	120 µg/mL	
**Moxalactam**		500–200 mg/kg//6–12 hr	100 µg/mL	
**Carbapenems**				
**Imipenem**	In high doses, neurological toxicity, seizures rarely occur. Hematological alterations, such as leukopenia, eosinophilia, or thrombocytosis, moderate and transient increases in transaminases, alkaline phosphatase. Doripenem is toxic by epidermal necrolysis and Steven-Johnson syndrome	1 g	69.9 mg/L	[215,216]
**Meropenem**		1 g	61.6 mg/L	
**Ertapenem**		1 g	164.6 mg/L	
**Doripenem**		500 mg	23 mg/L	
**Quinolones**				
**Pipemidic acid**	In some cases, tendinitis or tendon rupture. Fatal ventricular arrhythmias and neurotoxicity infrequently. Some quinolones that cause problems of phototoxicity (clinafloxacin), liver (trovafloxacin), or cardiac (grapafloxacin) toxicity have been withdrawn from the market	400 mg	4 mg/L	[217,218,219,220,221,222]
**Ciprofloxacin**		400 mg	1.6 mg/L	
**Ofloxacin**		400 mg	4 mg/L	
**Levofloxacin**		500 mg	5 mg/L	
**Oxazolidinone**				
**Linezolid**	Hematological toxicity, mitochondrial toxicity in blood cells and nerve fibers of the skin, hypoglycemia, lactic acidosis, and acute pancreatitis	1.5 mg/Kg	2.5 mg/L	[223,224,225,226]
**Lipopeptide**				
**Daptomycin**	Muscle toxicity. Neurological disorders (paraesthesia, dysesthesia) and eosinophilic pneumonia, skin and subcutaneous tissue disorders, hepatobiliary disorders, musculoskeletal, and connective tissue disorders.	4 mg/kg/day	62.4 µg/mL	[227]

**Table 3:** Biosensors used in the measurement of antibiotics.

Type of Biosensor	Antibiotic	Biosensor Characteristics	Matrix	Limit/Detection Range	Ref
**Electrochemical**	Aminoglycosides	RNA aptamers	Blood	2-6 µM	[241]
	Penicillin G	Gold NP, catalytic hydrolysis	Buffer	4.5nM	[242]
	Chloramphenicol and Kanamycin	Antibodies as bioreceptors	Buffer	45 pg/mL and 6.31 pg/mL	[24]
	Amikacin	Copper oxide modified carbon paste electrode	Buffer	1 µM	[243]
	Fluoroquinolones	Antibody modified magnetic beads	Milk	0.009 µg/L	[244]
	Tobramycin	RNA aptamers	Human serum	0.7 µM	[245]
	Ciprofloxacin	Antibodies on a poly (pyrrole-NHS) film	Buffer	10 pg/mL	[246]
	Penicillin	Capture—SELEX (DNA aptamers)	Milk	0.17 µg/L	[247]
		Magnetic graphene gold NP	Milk	0.057 ng/mL	[248]
	Tetracyclines	Carbon and oleic acid electrode antibodies	Milk	3.8 fM	[249]
	Ofloxacin	Automatic flow potentiometric system	Urine and serum	1 μM	[250]
**Optical**	Tetracycline Oxytetracycline Doxycycline	Fiber optic biosensor. Copolymer containing anthracene	Commercial samples and urine	1 μM 2 μM 2 μM	[251]
	Doxorubicin Daunorubicin	Fluorescence-induced LED fiber optic	Buffer	18 ng/mL 13 ng/mL	[23]
	Moxifloxacin	Hollow core photonic crystal fiber optic	Aqueous solution	682.43 ng/mL	[252]
	Vancomycin	Molecular imprinted polymer NP functionalized fiber optic grids (LPFG—MIP NPs)	Blood plasma	0.0032 ng/mL	[22]
	Sulfadimidine	Portable and reusable optofluidic-based biosensor platform	Dairy products	0.05 ng/L	[253]
	Tobramycin	Portable resonance plasmon setup (T-LSPR) coupled to DNA aptamers	Patient serum	3.4 µM	[254]
	Amoxicillin	SPR. Polymeric film (hydroxyethyl methacrylate-N-methacryloyl-(L) -glutamic acid)	Milk	0.0012 ng/mL	[255]
	Erythromycin	Fiber optic SPR/ERY printed nanostructure	Milk Honey	47.41 ng/mL 28.48 ng/mL	[256]
**SERS**	Sulfamethoxazole	Hydroxylamine stabilized silver NP	Enriched urine	1.7 µg/mL	[257]
	Ceftriaxone	Gold NP	Urine	0.7µM	[258]
	Ampicillin Penicillin G Carbenicillin Penicilloic acid	Hydroxylamine and silver NP	Deionized water	27 ng/mL 29 ng/mL 30 ng/mL 28 ng/mL	[259]
	Tetracycline	Raman fingerprint strip coated with anti-tetracycline mAb	Water	0.04 ng/mL	[260]
		Macroporous silicon and gold NP	Water	1 nM	[261]

NP: Nanoparticles, NHS: N-Hydroxysuccinimid, LPFG-MIP: Long Period Fiber Grating- Molecular Imprinted; Polymer SPR: Surface Plasmon Resonance, ERY: Erythromycin, SERS: Surface-enhanced Raman spectroscopy.
